# Interface Mechanism and Splitting Characteristics of Fiber-Reinforced Cement-Solidified Aeolian Sand

**DOI:** 10.3390/ma15082860

**Published:** 2022-04-13

**Authors:** Xiangdong Zhang, Shuai Pang, Jun Li, Xuefeng Zhang, Guanjun Cai, Lijun Tian

**Affiliations:** 1 School of Civil Engineering, Liaoning Technical University, Fuxin 123000, China; jwd101@126.com (X.Z.); lj0801@126.com (J.L.); zxf5105841@163.com (X.Z.); tianlijun2022@163.com (L.T.); 2 Beijing Jingneng Geo-Engineering Ltd., Beijing 102300, China; y651486373@126.com

**Keywords:** micro characterization, interfacial interaction, aeolian sand, splitting property

## Abstract

Experimental studies on reinforcing aeolian sand with cement and fiber are lacking, and the interface mechanism and splitting characteristics thus remain unclear. Herein, the interface mechanism and splitting characteristics of fiber-reinforced, cement-solidified, aeolian sand were experimentally assessed to investigate whether glass fiber exhibits better properties as a reinforcing agent than traditional fiber-free cement-solidified aeolian sand, and whether aeolian sand is applicable as a base material in geotechnical engineering. The splitting experiments involved the use of fiber-reinforced, cement-solidified aeolian sand samples that were differentiated based on the mixing schemes used to formulate them. Based on the strengthening control technology effects on the structural performance of the fiber-reinforced, cement aeolian, sand-mixed matrix material, the internal physical and chemical mechanisms of structural performance evolution were revealed and analyzed using scanning electron microscopy images. The experimental results show that the splitting strength of the sample reaches its maximum value at a combination of 6 mm glass fiber, 3‰ fiber, and 10% cement contents. In fiber-reinforced cement-solidified aeolian sand, cement hydrate forms more needle-shaped crystal products. The crystals adhere to the fiber surfaces that interweave with each other to form a porous and dense network. Although this improves the bonding force between the fiber and aeolian sand particles, the fibers are prone to fracture and slippage during the splitting process. The three-dimensional network structure formed by overlapping fibers is critical for the improvement of the splitting strength. The study’s findings will serve as benchmarks to achieve additional improvements in glass fiber-reinforced cement-solidified aeolian sand.

## 1. Introduction

Under the doctrine of “socialism with Chinese characteristics” implemented by the Chinese Communist Party, China’s economy has developed rapidly to become the second largest economy worldwide. Unfortunately, early steps in this historic economic development resulted in numerous problems, including ecological destruction and environmental pollution. These environmental problems have had a negative impact on development, and at various stages have affected people’s livelihoods and caused emotional and spiritual pain [1]. One notable example is the manner in which river sand, as a traditional building material, has caused serious damage to the ecological environment of rivers and mountains owing to its excessive exploitation. In recent years, government departments have prohibited the use of river sand. Therefore, the shortage of building materials has become a major problem that is currently restricting construction. However, aeolian sand, a sand alternative, is abundant in China, given that transportation due to wind erosion is the main cause of land desertification in the areas surrounding the Gobi Desert (approximately 16% of the country’s land area). At present, the geotechnical engineering application of aeolian sand focuses on mechanical compaction and grouting. Traditional curing treatment methods are problematic, with limited reinforcement ability, construction difficulties, and environmental pollution [2] being the current barriers to the uptake of aeolian sand for construction purposes.

Reinforcing mixed matrix materials for use in geotechnical engineering applications has been spearheaded by advances in the application of fiber reinforcement technology to soil; the latter involves the mixing of fibers with certain lengths with soil to form a new composite material that improves the tensile capacity relative to normal granular soil. Given the accessibility of numerous experimental studies on the mechanical properties of fiber-reinforced soil [3,4,5,6,7,8,9,10,11,12], many results exist that demonstrate an ability to control reliably material properties and responses. For example, Hongtao et al. [13] found that the strength of cement-improved soil increases as the fiber length and content increase. However, the strength decreased when the fiber content exceeded a specific limit. Qun et al. [14] found that the addition of fibers not only improved the ductility of cement soil, but also slowed down the crack propagation behavior. Qian [15] found that after the addition of basalt fiber to loess, the crack development speed of the sample is delayed, and the fiber on the fracture surface is pulled out, so the tensile strength of the sample is obviously improved. Hengchao et al. [16] found that an increase in fiber content and length led to an initial increase and to a subsequent decrease in the tensile strength of glass fiber-reinforced cement soil, with an optimum fiber content of 0.2% and an optimum fiber length of 6 mm. Hong et al. [17] obtained the variation law of the safety factor for slope stability based on consideration of parameters, such as the glass fiber length, glass fiber content, and slope height. Bo et al. [18,19] conducted a splitting strength test on polypropylene fiber-reinforced cement soil and found that an increase in fiber content resulted in increased splitting strength, tension–compression ratio, and ductility. Furthermore, from triaxial compression testing, it was found that adding polypropylene fibers could improve considerably the toughness of cement mortar soil. Wei et al. [20] used glass fiber for cement–soil reinforcement and concluded that when the contents of fiber with lengths of 6 mm or 12 mm were equal to 0.3%, the reinforcement effect of cement soil was the best. To elucidate the micromechanical characteristics of fiber-reinforced soil, Chaosheng et al. [21] used an electron microscope to scan and study the interface between fiber-reinforced plain soil and fiber-reinforced cement soil, and concluded that physical friction and hydration bonding between fiber and soil were the main mechanisms of the reinforcement effect. Deyin et al. [22] studied the interaction at the fiber–soil interface of unsaturated cohesive soil reinforced by polypropylene fiber using scanning electron microscopy (SEM), and concluded that fiber reinforcement was mainly achieved by the one-dimensional (1D) reinforcement of single fibers, and three-dimensional (3D) reinforcement of fiber webs. Lei et al. [23] used SEM and showed that when the fiber content was 0.25%, the fiber dispersed evenly in the soil. This even dispersion played a key role in enabling a 3D fiber web to be formed. The soil produced a gripping effect on the fiber, and the main failure modes of the fiber were slip and wear. Tang et al. [24,25] found that after the fiber was added to the soil, friction and cohesive force were generated at the interface between the fiber and the soil, and the magnitude of this net interfacial force directly affected the improvement effect of the fiber on the soil owing to the generated interfacial shear strength. Shizong [26] analyzed the principle of cement soil strength enhancement by the fiber, and asserted that the possible mechanisms can be summarized according to the “bending mechanism” and “interweaving mechanism” concepts. To date, studies of the microscopic properties of fiber-reinforced soil have been limited because the soil types considered were loess, clay, soft soil, or expansive soil. Only a few research studies have been conducted on aeolian sand, especially curing aeolian sand with glass fiber and cement as reinforcing materials. Given the complex interaction between geosynthetics, findings of fiber reinforcement using other soils cannot be extended to aeolian sand. Therefore, conducting experimental studies to establish an evidence base specific to the reinforcement of aeolian sand with cement and fiber is necessary.

In this study, aeolian sand is adopted to fill roadbeds in desert areas as the engineering application of interest, and aeolian sand in the Zhangwu area of Liaoning province is the base material with research interest. A cross-scale research method that combines macroscopic splitting mechanical tests with SEM technology has been adopted for this study to explore the influence of different fiber blending schemes on the splitting strength of cement-fiber-solidified aeolian sand. Based on the basic theory of interface mechanics [27], The effect of additive (glass fiber, cement) content and fiber length on the splitting strength and fiber morphology of cement-fiber solidified aeolian sand under splitting condition and the mechanisms of the cement–fiber solidified aeolian sand reinforcement–soil interface are revealed. The research shows that the engineering mechanical properties of aeolian sand can be improved by adding appropriate amounts of cement and fiber, and the research results can provide a scientific basis for the prevention and treatment of subgrade diseases of cement-fiber solidified aeolian sand.

## 2. Materials and Methods

### 2.1. Test Materials

**Figure 1:**
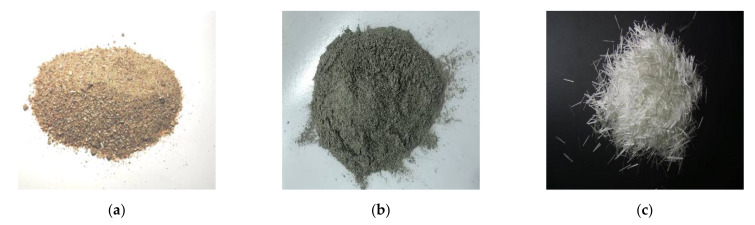
Tested raw materials. (**a**) Aeolian sand; (**b**) Cement; (**c**) Glass fiber.

To prepare samples of fiber-reinforced cement-solidified aeolian sand, raw materials, such as aeolian sand, cement, and glass fiber, should be prepared in advance, as shown in Figure 1. The matrix material used in the experiment is aeolian sand from the Zhangwu area of the Liaoning Province. The gradation of this natural aeolian sand is poor, the particle sizes are in the 0.63–0.92 mm range, unevenness coefficient Cu = 3.74 (unevenness coefficient Cu < 5 indicates poor grain gradation), and the curvature coefficient Cc = 0.82 (curvature coefficient Cc < 1, which indicates poor grain gradation). The aeolian processes of transportation due to wind erosion have a rounding effect on the particles, such that their surfaces are relatively smooth. The basic physical and mechanical parameters of aeolian sand are listed in Table 1 [28]. The cement used for sand consolidation was ordinary Portland cement P.O 42.5. Glass fiber was produced by Jiangxi Yuanyuan New Material Company, and its physical and mechanical properties are listed in Table 2.

### 2.2. Test Scheme

#### 2.2.1. Compaction Test

**Figure 2:**
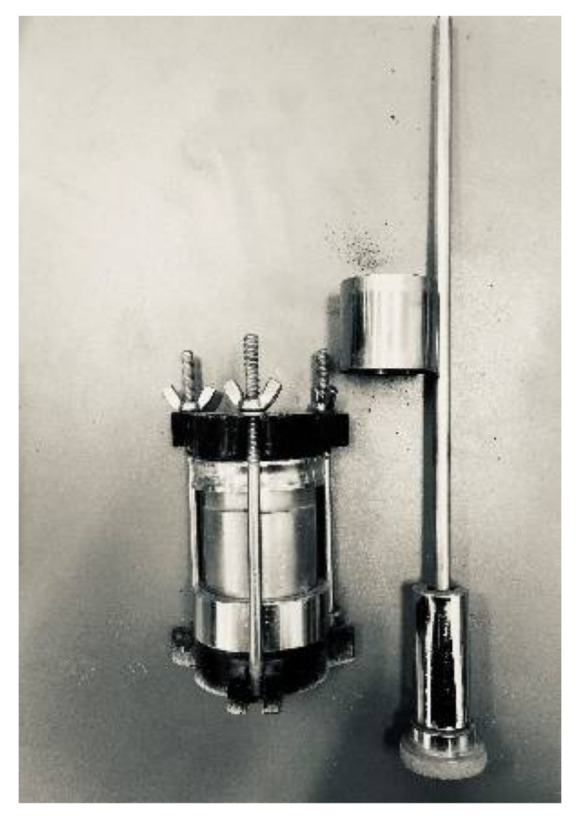
Compaction instrument.

**Figure 3:**
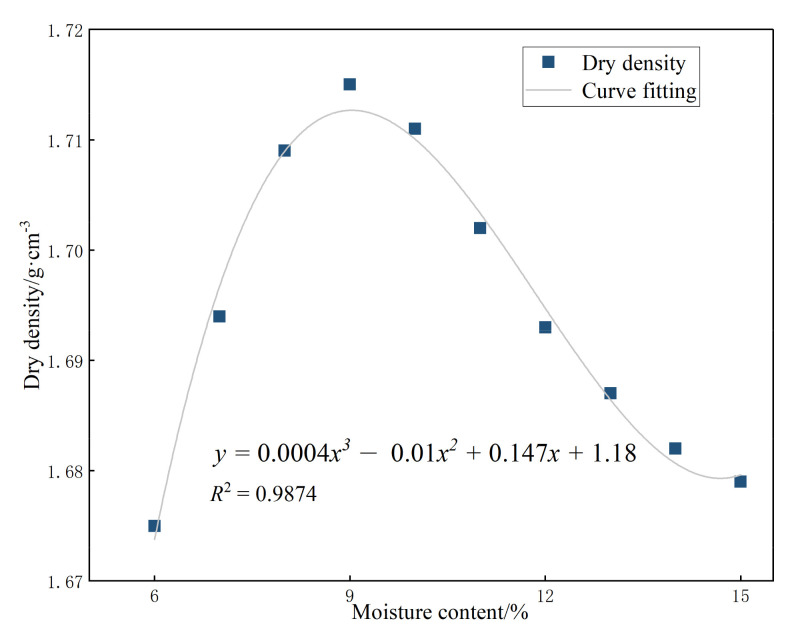
Compaction curve of solidified aeolian sand.

Before the test, the aeolian sand was dried and mixed with cement and glass fiber using the amount of water required to attain the predetermined moisture content. The mixture was then placed into the compaction cylinder in a layer-by-layer manner. After each layer (five layers in total) was laid, the mixture was hammered 20 times with a compaction hammer from the same falling distance. The mixture was finally filled into the compaction cylinder, as shown in Figure 2. The dry density can be calculated as,

(1) $$\rho_{d}=\frac{\rho }{1+\omega }$$

where ρ represents the density of the sample and ω is the moisture content of the sample. The compaction curve derived from Equation (1) is shown in Figure 3, and the mixture of cement and aeolian sand has an optimal moisture content of 9% and a maximum dry density of 1.718 g/cm^3^.

#### 2.2.2. Sample Preparation and Testing

Based on the research by Hengchao et al. [16], the current study controlled the factor values to the following settings for sample preparation: (i) the contents of glass fiber were 2‰, 3‰, 4‰, and 6‰ of aeolian sand, (ii) the fiber lengths were set at 3 mm, 4.5 mm, and 6 mm, (iii) the cement contents were set at 6%, 8%, and 10% of aeolian sand, and (iv) the optimal moisture content at 9%. Glass fiber, cement, aeolian sand, and water were evenly mixed and poured into a three-valve saturator mold in five layers, and each layer was scraped and compacted 20 times to make a standard cylindrical sample with the dimensions of Φ39.1 mm × 80 mm.

Three samples were prepared for each group, and the orthogonal design method was used for qualitative analysis. The interaction between factors was not considered on this occasion. Table 3 lists the levels used for the orthogonal design test factors. Table 4 lists the orthogonal test results and range analysis using the orthogonal design assistant, while Table 5 lists the range analysis results.

From the range analysis outcomes of the orthogonal test (see Table 5), it is concluded that the primary and secondary degrees of influence on the splitting strength are ranked in the following (descending) order: cement content, fiber length, and fiber content. The method of controlling variables by local factors was used for quantitative analyses. Table 6 summarizes the test scheme, which consists of 13 groups of proportions. Three samples were prepared for each group, which resulted in a total of 39 samples.

**Figure 4:**
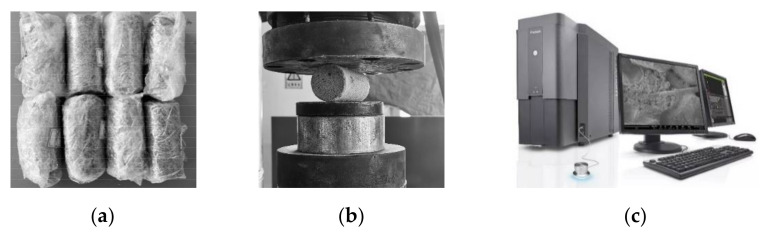
Sample preparation and test process. (**a**) Sample preparation and maintenance; (**b**) Splitting loading process; (**c**) Scanning electron microscopy (SEM).

The prepared samples were wrapped with a preservative film, as shown in Figure 4a. According to the conclusions drawn in a previous study [16] that considered soil modified by glass fiber cement reinforcement, (i) the splitting tensile strength increases as a function of the curing age, (ii) the growth rate of splitting tensile strength decreases as a function of the curing age, and (iii) the growth rate of splitting tensile strength decreases abruptly and gradually flattens after 14 days (14 d) of curing. Therefore, in this test, the conditions set for 14 d of curing in the standard curing box were temperature: 20 °C and humidity: 90% (both of these were maintained by their respective controllers). Furthermore, this test used a TAW-2000 electrohydraulic, servo rock, triaxial apparatus (The equipment is produced by Changchun Chaoyang Test Instrument Company) to perform splitting loading on the sample which reached the curing age, as shown in Figure 4b, at a controlled loading rate set at 0.5 mm/min. The maximum force at which the sample failed, P, was recorded. At the conclusion of the cleavage test, the samples were scanned using a Phenom XL scanning electron microscope on a Vernier bench, as shown in Figure 4c, to obtain the microscopic morphology of the samples.

## 3. Splitting Test Results and Analysis

### 3.1. Analysis of Section Failure Form

**Figure 5:**
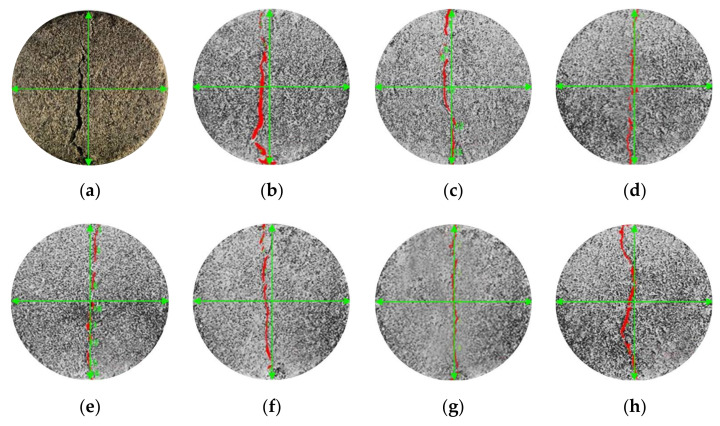
Failure specimen modes. (**a**) Section map; (**b**) None; (**c**) 3‰, 3 mm; (**d**) 3‰, 4.5 mm; (**e**) 2‰, 6 mm; (**f**) 3‰, 6 mm; (**g**) 4‰, 6 mm; (**h**) 6‰, 6 mm.

Within the range of experimental study, record the force on the sample mixed with 3‰ 6 mm fiber when it fails, and then apply the same force to the samples under different conditions to observe the failure modes of all samples, as shown in Figure 5. Figure 5a shows the failure form of fiber-free, cement-cured, aeolian sand samples after the splitting test. After noise reduction, enhancement, segmentation, recognition, and measurement using Image-Pro Plus digital image processing technology, the processed images shown in Figure 5b–h are obtained, and the characteristic shapes of cracks after processing can be clearly observed.

Figure 5 shows that when the same pressure was applied, there was little difference in the splitting failure form between the samples of fiber-free cement-solidified aeolian sand and fiber-reinforced cement-solidified aeolian sand. Figure 5b shows the shape of the fiber-free, cement-solidified, aeolian sand sample when it was destroyed in the splitting test. It took a short time to achieve failure, and the crack expanded rapidly; after the failure, a significant through crack appeared in the middle that justifies brittle failure characteristics. Figure 5c–h show the failure modes of fiber-reinforced cement-solidified aeolian sand samples after splitting test loading. This shows that adding glass fiber into cement-cured aeolian sand can improve the crack propagation resistance, effectively enhancing the energy absorption capacity in the deformation process and increasing the deformation resistance. Thus, the cement–fiber-cured aeolian sand yields a specific toughness, and the original brittle failure mode is transformed into an early ductile failure mode, thus justifying the description of these aeolian sand samples as cement-solidified and fiber-reinforced.

### 3.2. Analysis of Splitting Strength Test Results

**Figure 6:**
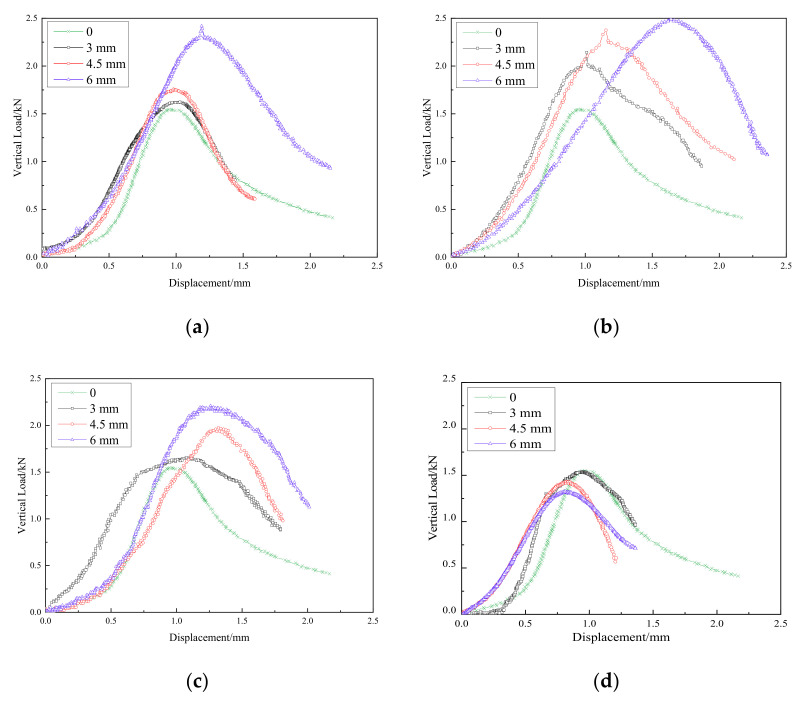
Vertical load–displacement curves of specimens. (**a**) Fiber content 2‰; (**b**) Fiber content 3‰; (**c**) Fiber content 4‰; (**d**) Fiber content 6‰.

The relationships between axial load and displacement during splitting loading for 10% cement content at different fiber contents (0‰, 2‰, 3‰, 4‰, and 6‰) and fiber lengths (3 mm, 4.5 mm, and 6 mm) are shown in Figure 6.

**Figure 7:**
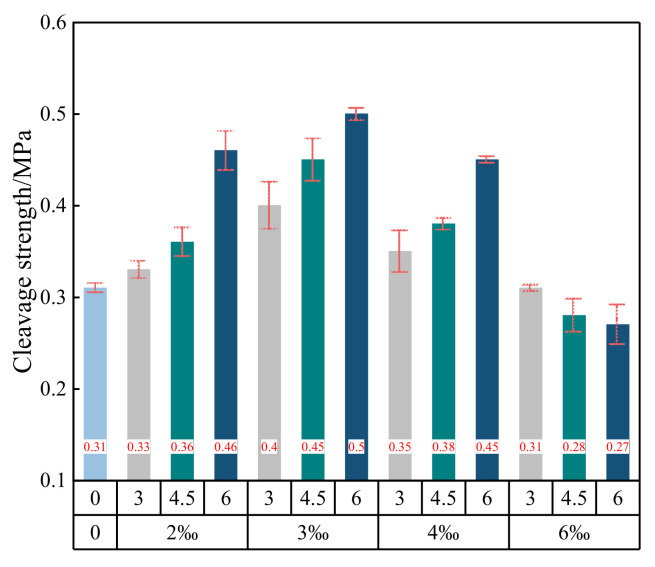
Change of splitting strength with fiber.

The load corresponding to the peak point in Figure 6 can be taken to be the maximum splitting load, Pmax, that the sample can bear. According to the theory of elasticity [29], the splitting strength of the cylindrical sample can be determined as.

(2) $$\sigma_{t}=\frac{2p_{max}}{\pi ld}$$

where l is the thickness of the sample and d is the diameter of the sample. The relationship between the average splitting strength can be determined by Equation (2) and the fiber content and length are shown in Figure 7.

It can be seen from Figure 7 that when the fiber content is greater than 3‰, the splitting strength of the fiber-reinforced cement-solidified aeolian sand samples gradually decreases as a function of the fiber content. After 6‰ of fibers with lengths of 6 mm is added, the splitting strength is 0.27 MPa; this is 0.23 MPa lower than that of fiber samples (lengths equal to 6 mm) with a content of 3‰ (0.50 MPa). After the addition of fibers with a content of 6‰ (lengths of 4.5 mm), the splitting strength of the sample becomes 0.28 MPa, which is 0.17 MPa lower than that of the 4.5 mm fiber sample with a fiber content of 3‰ (0.45 MPa). After the addition of fibers (content: 6‰, lengths: 3 mm), the splitting strength of the sample is 0.31 MPa, which is 0.09 MPa lower than that of fiber samples with a content of 3‰ and with lengths of 3 mm (0.4 MPa). This is attributed to the fact that when the glass fiber content is too high, it is difficult for the glass fiber to be evenly stirred, thus resulting in agglomeration inside the sample. The agglomerated fiber cannot be cemented inside the aeolian sand and cannot fully exert its tensile effect. Instead, it weakens the gripping force between the aeolian sand particles and the glass fiber, resulting in a decrease in the overall splitting strength of the sample.

When the fiber content is small (2‰, 3‰, and 4‰), the splitting strength increases as the fiber length increases. When the fiber content is large (6‰), the splitting strength decreases with an increase in the fiber length. When the fiber content is 3‰ and the length is 6 mm, the splitting strength reaches the maximum (0.50 MPa), which is 0.19 MPa higher than that of the fiber-free cement-cured aeolian sand sample (0.31 MPa). The splitting strengths of the fiber-reinforced cement-cured aeolian sand samples with fiber lengths of 3 mm and 4.5 mm are 0.09 MPa and 0.14 MPa, respectively; these are higher than that of the fiber-free cement-cured aeolian sand. In the literature [16], scholars have studied the splitting tensile strength of glass fiber cement improved soil. The research shows that with the increase of fiber content, the splitting tensile strength of the sample first increases and then decreases, with the optimal content of 2‰ and the optimal fiber length of 6 mm, which is basically consistent with the law obtained in this paper. Fatahi et al. [30] studied the influence of polypropylene fiber, carpet fiber, and steel fiber on the compressive strength and tensile strength of cement–soil with soft soil as the test object. The test results show that the splitting tensile strength of fiber cement–soil increases with the increase of carpet fiber content and steel fiber content. When the polypropylene fiber content is small, the fiber has little effect on the splitting tensile strength of fiber cement–soil. Yadav et al. [12] used NaOH-treated coconut fiber, pool ash produced by thermal power plant and cement to improve clay. Through the splitting tensile test, it was found that the splitting tensile strength first increased and then decreased with the increase of fiber content, and reached the maximum when the fiber content was 1%.

### 3.3. Analysis of Fiber Reinforcement Effect

To evaluate the contribution of glass fiber to the splitting strength of fiber-reinforced, cement-solidified aeolian sand, the condition of constant cement content is imposed, and the reinforcement effect coefficient of the splitting strength is evaluated as:

(3) $$R_{tf}=\frac{\sigma_{\mathrm{tf}}-\sigma_{t}}{\sigma_{t}}\times 100\;\%$$

where $R_{tf}$ is the splitting strength reinforcement effect coefficient, $\sigma_{\mathrm{tf}}$ is the splitting strength of cement–fiber-solidified aeolian sand (MPa), and $\sigma_{t}$ is the splitting strength of aeolian sand solidified by the cement without fiber (MPa).

According to Equation (3), the splitting strength reinforcement effect coefficient of different fiber mixing schemes for fiber-reinforced cement-solidified aeolian sand was calculated, and the calculated results are shown in Table 7.

It can be observed from Table 7 that when the fiber content is 2‰, 3‰, and 4‰, the splitting strength reinforcement coefficient of cement–fiber-solidified aeolian sand samples at different fiber lengths gradually increase as the fiber length increases. The fiber reinforcement effect was the most obvious when the sample was mixed with 3‰ 6 mm glass fiber, which shows that the splitting strength of cement-fiber-solidified aeolian sand can be improved to varying degrees by adding glass fiber. However, when 4.5 mm and 6 mm glass fibers are used and the fiber content is 6‰, the reinforcement effect coefficient is negative, which shows that the splitting strength of the sample is weakened at this time because of the large fiber content, which plays a negative role. From the overall analysis of the data in Table 7, the contribution increment of the splitting strength in the fiber-reinforced cement-solidified aeolian sand varies dynamically. With the change in blending scheme, the weight of the contribution of the fiber to the splitting strength of fiber-reinforced cement-solidified aeolian sand is different, such that there is an optimal value, which is related to the fiber content, fiber length, and other factors.

## 4. Interface Mechanism of Fiber-Reinforced Cement-Solidified Aeolian Sand

### 4.1. Overall Morphological Characteristics

Collect the sample crack position for SEM. The microstructure of fiber-reinforced cement-solidified aeolian sand samples with different mixing schemes after splitting failure was observed with the use of SEM. When typical areas were observed, the low-magnification mode was switched to high-magnification mode, the local areas were enlarged, and representative images were selected to facilitate acquisition and storage. The magnification of the SEM images obtained in this experiment ranged from 100 to 1000×.

**Figure 8:**
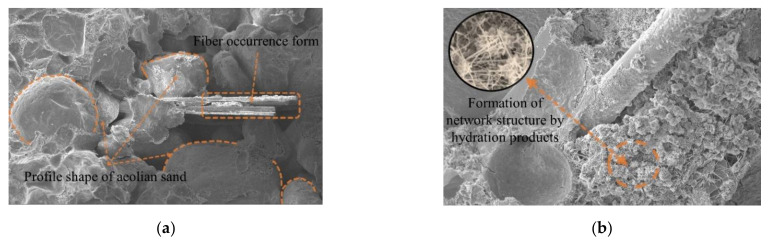
Scanning pictures of cement-fiber-solidified aeolian sand with SEM. (**a**) Microscopic scanning image of sample (100×); (**b**) Microscopic scanning image of sample (1000×).

When the magnification is 100 times, the scanning image of the electron microscope is shown in Figure 8a. The image clearly shows the outline of aeolian sand particles, the pores between particles, and the existing forms of fibers in cement-cured aeolian sand. When the magnification is 1000×, the scanning image of the electron microscope is shown in Figure 8b, which clearly shows the damaged form of a single fiber in fiber-reinforced cement-solidified aeolian sand and the contact interface structure between cement hydration products and aeolian sand particles.

**Figure 9:**
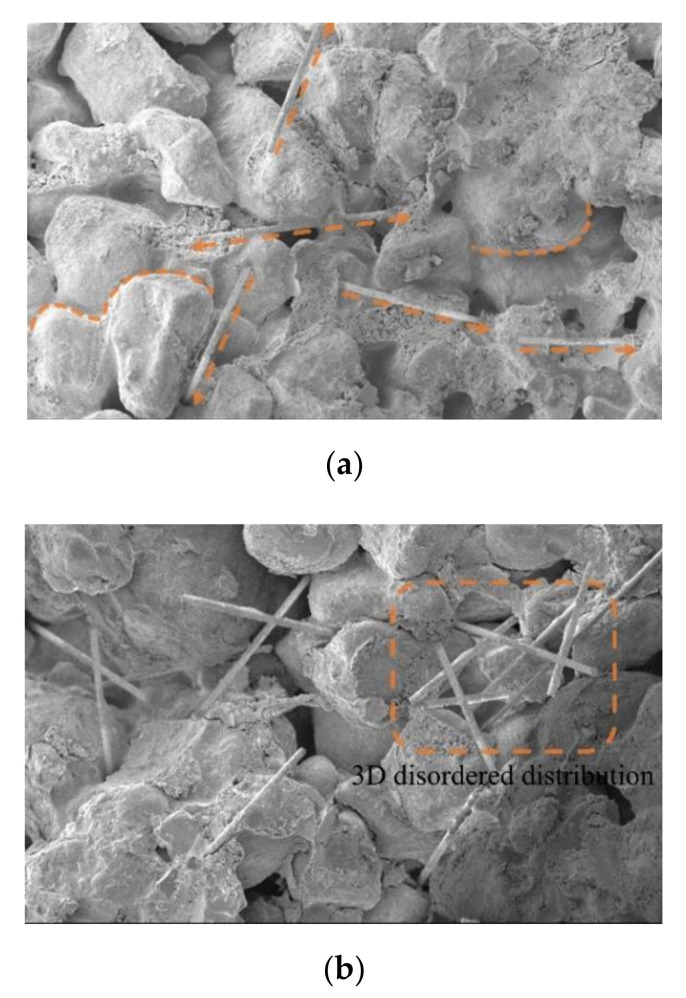

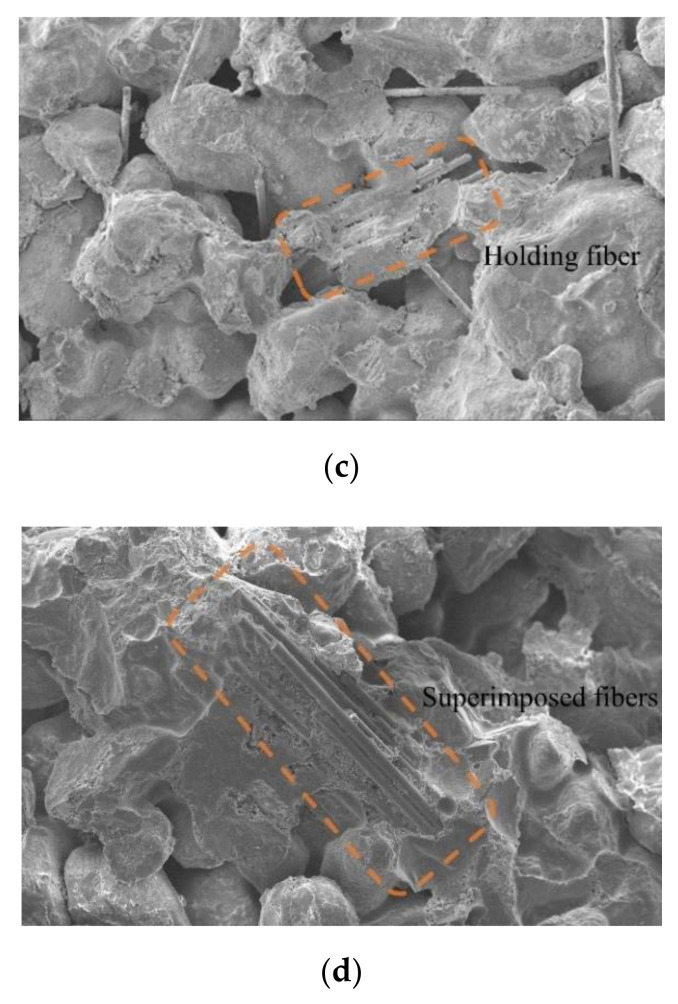
Microstructure of cement-fiber-cured aeolian sand. (**a**) SEM images of 6 mm fiber samples doped at 2‰ (100×); (**b**) SEM images of 6 mm fiber samples doped at 3‰ (100×); (**c**) SEM images of 6 mm fiber samples doped at 4‰ (100×); (**d**) SEM images of 6 mm fiber samples doped at 6‰ (100×).

**Figure 10:**
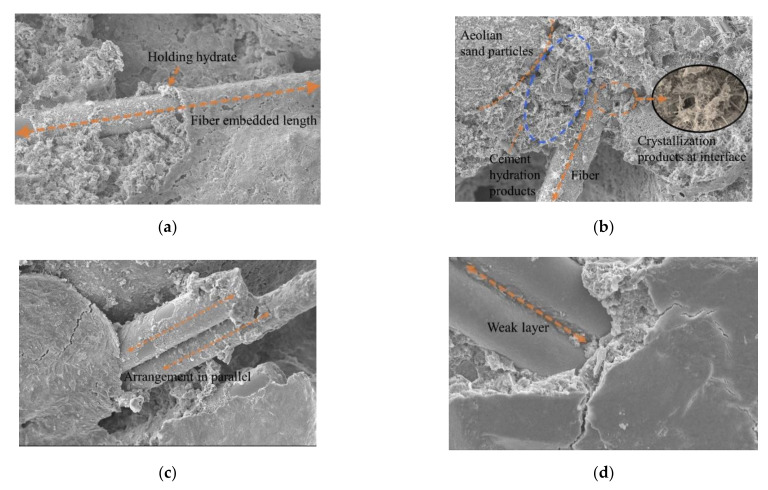
Microscopic characterization of fiber-reinforced cement-solidified aeolian sand. (**a**) Image of single-fiber gripping effect (1000×); (**b**) Bond image of steel-sand interface (1000×); (**c**) Image of parallel arrangement of fibers (1000×); (**d**) Weak layer after fiber superposition (1000×).

SEM images of aeolian sand mixed with 2‰ to 6 mm fibers are shown in Figure 9a. It is evident that aeolian sand particles are round in outline, the sand particles retain their roundness owing to transportation due to wind erosion, and most of them are approximately ellipsoidal. There is no obvious difference between aeolian sand particles, and most of them are in face-to-face contact, while glass fiber was mixed with cement aeolian sand matrix by inserting and embedding, which is randomly distributed and has a high degree of dispersion. At the initial stage of curing fiber-reinforced cement-solidified aeolian sand samples, the fibers and aeolian sand particles are combined by extrusion and wrapping. During the hydration reaction, the cement hydrate grows continuously, filling the gaps between the aeolian sand particles and between the fibers and aeolian sand particles, as shown in Figure 10b. This generates a cohesive force at the contact interface, thus forming an effective bond that joins the fibers and aeolian sand particles together. Owing to the low-fiber content, the fiber and cement aeolian sand were wrapped by a single fiber, and the fiber in the sample was tightly wrapped by the surrounding aeolian sand particles, as shown in Figure 10a. With an increase in the embedding length of the fiber, the wrapping area increased. When the sample is deformed or destroyed by an external load, the fiber is in a tensile state, and the bonding and friction forces between the fiber and the aeolian sand particles limit the extent of sliding relative to each other, which plays a connecting role and reduces the relative displacement.

Figure 9b shows the scanning image of the electron microscope when the fiber content is increased to 3‰. The fibers were evenly distributed randomly in the sample (crisscrossed) with shapes similar to those of the roots embedded in the soil, and thus formed a relatively sTable 3D spatial structure system inside the fiber-reinforced, cement-solidified, aeolian sand mixed matrix. When the aeolian sand particles are dislocated and rearranged by an external load, the relatively stable fiber net structure system restricts the displacement and deformation of aeolian sand particles, which can better transmit the external load to other areas inside the sample. This helps reduce stress concentration, realizes the effect of sharing stress, and improves the response of the entire system. The hydration reaction forms many needle-like crystal products between the aeolian sand particles and between the aeolian sand particles and fibers; accordingly, crystals adhere to the surface of the fibers. As shown in Figure 10b, hydration products interweave with each other to form a porous and dense network structure, which not only improves the cohesive force between the fibers and aeolian sand particles, but also enhances the interface between them. In addition, the glass fiber is relatively soft, and the cement hydrate crystal is wrapped on the fiber surface in a manner similar to the hard shell case, thus forming an anchoring area around the fiber. This not only improves the stiffness of the fiber but also glues the discrete fibers into a net-like, whole structure, which plays a major role in bonding and stabilizing the relative position between the aeolian sand particles and the fiber in heterogeneous materials. Thus, it is not easy for the fiber and the aeolian sand particles to slide relatively to each other when they are loaded, which effectively inhibits the formation and development of cracks and enhances the cement-fiber solidification.

Figure 9c shows the scanning image of the electron microscope when the fiber content was increased to 4‰. The fibers were not completely dispersed inside the sample, and the degree of dispersion was reduced. The fibers were arranged in parallel among the aeolian sand particles. As shown in Figure 10c, agglomeration occurred, and the cement hydrate existing among the particles was reduced. Some of the fibers are not in full contact with the aeolian sand particles, thus resulting in the reduction of the gripping area between the aeolian sand particles and the fibers. When the fiber content is increased to 6‰, the scanning image of the electron microscope is shown in Figure 9d, and the fibers in the cement–fiber-solidified aeolian sand sample form a cluster, thus resulting in a serious phenomenon of fiber superposition; an interface of the weak layer can arise from fiber superposition, as shown in Figure 10d. As the cement hydration reaction progresses, hydrates cannot fill the gap between particles and fibers. Additionally, there is no cementation between aeolian sand particles and fibers, which loosens the matrix of aeolian sand, and the overall performance is poor. When the sample is subjected to an external load, the fiber with a weak layer would be rapidly damaged by a sliding crack. Accordingly, the fiber cannot be used for reinforcement. Thus, the entire structure of the sample would be rapidly damaged.

### 4.2. Analysis of Failure Mode of Reinforcement-Sand Interface

**Figure 11:**
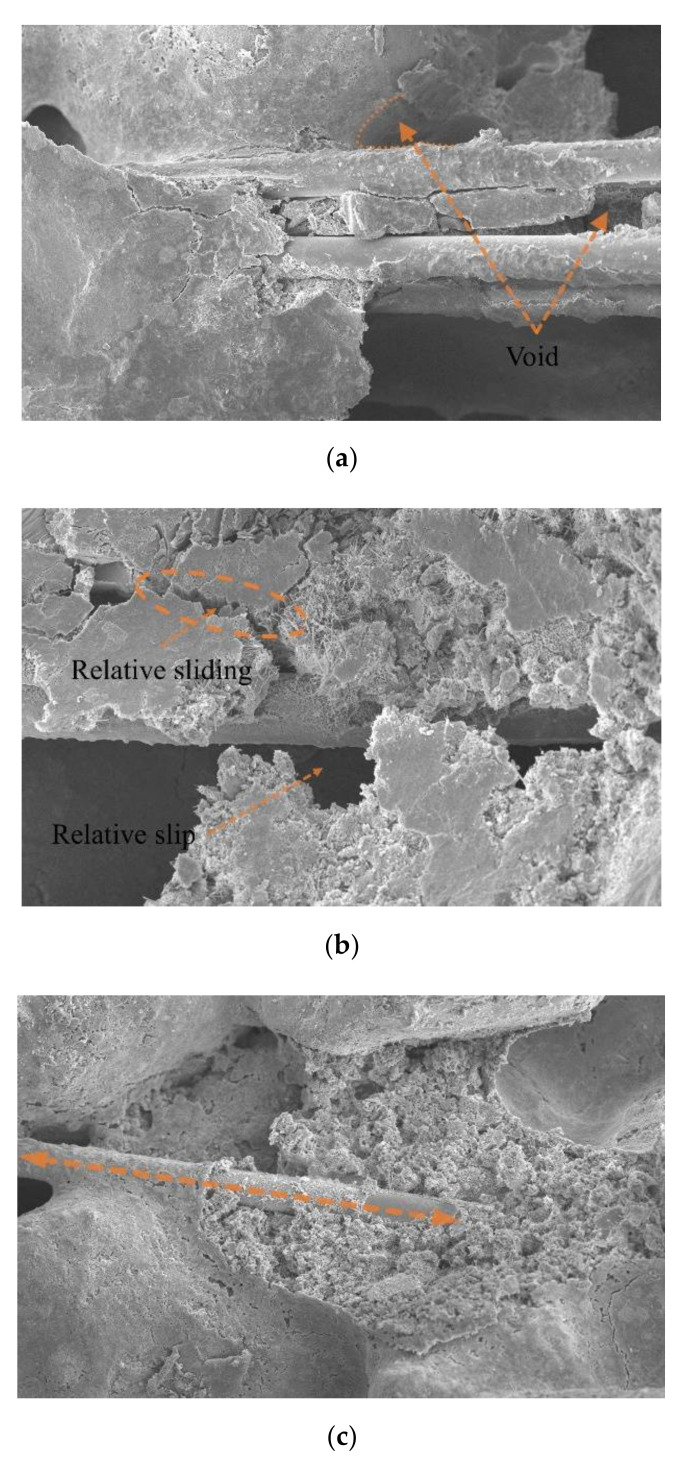

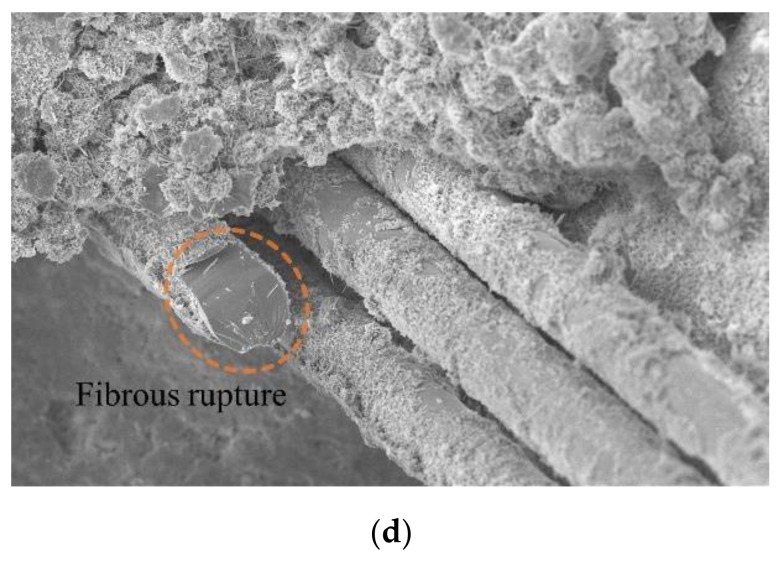
Fiber failure modes. (**a**) Image of the bond condition between the fibers and sand (500×); (**b**) Slip and dislocation at the interface between the reinforcement and sand (1000×); (**c**) Image of the bond condition between the fiber and sand (500×); (**d**) Fiber breakage (1000×).

Images of cracks in the fiber-reinforced cement-solidified aeolian sand samples were obtained by SEM. Observation of the cracks after the splitting test showed that the deformation and failure modes of fibers in the aeolian sand action area were mainly divided into two types: the relative slip of the contact interface between the glass fibers and aeolian sand particles and the fiber breakage, as shown in Figure 11. It can be clearly observed that the bonding condition of the mixture of fiber and cement aeolian sand is not perfect, and there are gaps around it. Pore water on the interface plays a lubricating role and reduces the interfacial friction and cohesion between the fiber and aeolian sand. Therefore, when the fiber is subjected to an external load, the fiber overcomes the friction and cohesion between its surface layer and aeolian sand particles, and the friction between aeolian sand particles. Together with the surrounding aeolian sand particles, it exhibits an obvious dislocation and a relative slip, as shown in Figure 11b. However, its overall shape is still relatively complete.

Figure 11c shows that the fiber surface layer is wrapped with a large amount of mixture of cement hydrate and aeolian sand, and the bonding between them is in good condition. When subjected to an external load, the fiber remains in an elastic working state, and its deformation increment evolves with the change in the macro stress acting on the surface of the fiber-cement solidified aeolian sand sample. When the interaction force between the fiber and the mixed matrix interface is greater than the tensile strength of the fiber, the fiber no longer provides an incremental contribution to the macro stress on the surface of the sample, but breaks, as shown in Figure 11d.

**Figure 12:**
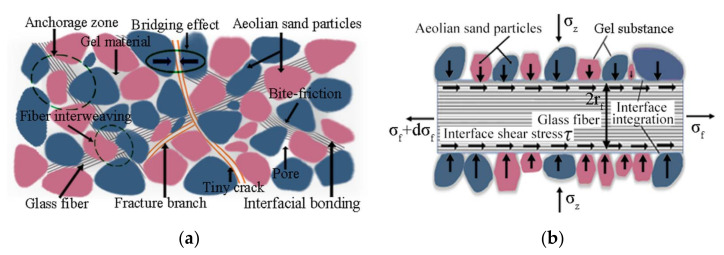
Interface action diagram of cement-fiber solidified aeolian sand. (**a**) Interface model of cement-fiber-solidified aeolian sand; (**b**) Force diagram of fiber unit.

For the fiber-reinforced, cement-solidified aeolian sand composite material, the complex composition of glass fiber, cement and aeolian sand material determines the heterogeneity and variability of physical and mechanical properties of the material. Based on the ideal form of fibers (which assumes that glass fibers are randomly distributed in the mixed matrix of cement aeolian sand approximately uniformly), an interface model of fiber-reinforced, cement-solidified aeolian sand is established to clarify the microphysical and mechanical mechanism of fiber reinforcement effect, as shown in Figure 12a. When the fibers are uniformly distributed in the mixed matrix of cement-cured aeolian sand, it forms a spatial network structure similar to a “magpie nest.” The fibers overlap with each other, and there are many intersections, surrounded by aeolian sand particles. In this study, the glass fiber is assumed to be a linear 1D cylindrical element, and its stress analysis is conducted as shown in Figure 12b.

On that glass fib micro unit with length dl, the equilibrium equation can be obtained from the interaction between aeolian sand and glass fib as follows,

(4) $$\pi r_{f}^{2}\left(\sigma_{f}+d\sigma_{f}\right)=\pi r_{f}^{2}\cdot \sigma_{f}+2\pi r_{f}\cdot \tau \cdot dl$$

where $\sigma_{f}$ is the axial stress of the fiber, τ is the shear stress at the interface between the aeolian sand particles and fibers, and $r_{f}$ is the fiber section radius (mm). By simplifying the equilibrium equation, we obtain

(5) $$\frac{d\sigma }{dl}=\frac{2\tau }{r_{f}}$$

Assuming that the bonding between the glass fiber and aeolian sand is divided into two parts, namely mechanical friction and cement cementation, the generalized Mohr–Coulomb strength criterion can be used to express the shear stress τ at the interface between aeolian sand particles and fibers:

(6) $$\tau =\sigma_{z}\tan\phi +c$$

where $\sigma_{z}$ represents the positive pressure of contact between sand and fiber, which is related to the degree of compaction of the improved aeolian sand, ϕ denotes the friction angle of contact between sand and fiber and represents the roughness of the contact surface, and c indicates the cohesion of contact between sand and fiber, which is related to the degree of cement hydration reaction.

Substituting Equation (6) into Equation (5) and integrating the two sides yields

(7) $$\sigma =\int d\sigma_{f}=\frac{2}{r_{f}}\left(\sigma_{z}\int_{0}^{l}tan\phi dl+\int_{0}^{l}cdl\right)$$

Assuming that the cohesion and internal friction angle of the interface between the fiber and sand are uniformly distributed on the entire fiber, Equation (7) can be simplified as

(8) $$\sigma =\frac{2l}{r_{f}}\left(\sigma_{z}tan\phi +c\right)$$

Subject to the condition of the splitting experiment, the failure modes inside the fiber-reinforced, cement-solidified, aeolian sand are as follows:

(a) Failure mode I: when the glass fiber is too short, the reinforcing and toughening effect of the fiber on the cement-cured aeolian sand is weak, and the external force is resisted only by the strength of the cement-cured aeolian sand itself
(b) Failure mode II: fiber and sand slip, and the mechanical friction between the fiber and sand interface only provides strength
(c) Failure mode III: Cement hydration products bind sand particles and fibers together. Subject to the action of splitting tensile load, sand particles first break and separate, and the exposed fibers stretch subject to the action of an external load. When the critical tensile stress of the fibers is reached, the fibers are broken.

The failure mode of the glass-fiber-solidified soil is closely related to the length of the fiber. It can be observed from Equation (8) that the tensile stress $\sigma_{f}$ at both ends of the glass fiber is closely related to the fiber type, which is proportional to the fiber length and inversely proportional to the fiber section radius. When the stress σ at both ends of the fiber reaches the ultimate tensile strength $\sigma_{f}$ of the fiber, the critical length of the fiber is

(9) $$l_{c}=\frac{\sigma_{f}}{2\left(\sigma_{z}tan\phi +c\right)}r_{f}$$

The criterion of fiber failure mode is as follows,

(10) $$\left\{\begin{array}{c} l<l_{c},Failure\;mode\;I\\ l>l_{c},Failure\;mode\;\mathrm{II} \end{array}\right.$$

The interfacial interaction effect between glass and aeolian sand particles is the internal mechanism based on which fibers improve the toughness and splitting strength of cement aeolian sand. According to Tang et al. [21], the mechanical interaction between the reinforcement and soil interface includes bonding and friction. In this study, it is assumed that the interface interaction between the fiber and aeolian sand particles consists of two parts: interface friction and cohesion generated by the gel material. The size of the interface friction is mainly related to the effective contact area between the fiber and aeolian sand particles, water content, and other factors, while the cohesion generated by the gel material is mainly influenced by the cement’s content.

Subject to the action of an external load, the relative movement between the glass fiber and aeolian sand particles makes the fiber tensile because there is a certain difference between their elastic moduli. Because of the good tensile property of the glass fiber, it can share a part of the external load for aeolian sand particles. When the sample reaches the ultimate tensile strength locally, microcracks begin to appear on the surface, the aeolian sand around the microcracks no longer bore the tensile stress, and the horizontal tensile stress inside the sample is generated by the fiber. At this time, the tensile properties of the fiber play a major role. Furthermore, the “anchoring area” can effectively restrain further development of the existing tensile crack and continue to bear part of the tensile stress in the load in the crack until the fiber reaches the ultimate tensile strength. The sample is subjected to yield failure. For example, when the cement–fiber solidified aeolian sand blend material is mixed with 6 mm glass fiber, such as 3‰, the splitting strength of the sample is increased by 61.29% on the basis of cement aeolian sand material without fiber. Therefore, adding an appropriate amount of glass fiber can transform the failure form of the sample from brittle to plastic. To some extent, the formation and development of internal small cracks is delayed, which is conducive to the improvement of the overall stability of the sample. The research results can provide a scientific basis for the prevention and treatment of cement–fiber, solidified aeolian sand roadbed diseases.

## 5. Conclusions

Based on the splitting test and micro-electron microscope scanning test of fiber-reinforced cement-solidified aeolian sand samples at different mixing schemes, the corresponding splitting strengths and different microcharacterizations of the fiber-sand bonding interface were obtained, and the following conclusions were drawn by analyzing the test results:

(1) Adding an appropriate amount of glass fiber can effectively improve the splitting strength of cement-cured aeolian sand. When the fiber content was 3‰ and the fiber length was 6 mm, the fiber forms in the sample are ideal, which can increase the splitting strength of cement-fiber solidified aeolian sand, and the reinforcement effect coefficient of the splitting strength reached the maximum value.
(2) In cement-fiber-solidified aeolian sand, cement hydration forms more needle-like crystal products, which adhere to the surface of the fiber and interweave with each other to form a porous and dense network structure. This can effectively improve the cohesive force between the fiber and aeolian sand particles. The interfacial interaction effect between glass and aeolian sand particles is the internal mechanism based on which fibers improve the toughness and splitting strength of cement aeolian sand.
(3) The reinforcement mechanism of the fiber is mainly a result of the gripping effect of a single fiber and the fiber web formed by the random distribution of fibers. During the splitting process, fibers are prone to fracture and slip. When the sample is damaged, some fibers are constantly cracked, thus improving the failure toughness of the sample. Adding an appropriate amount of glass fiber can transform the failure form of the sample from brittle to plastic. To some extent, the formation and development of internal small cracks is delayed, which is conducive to the improvement of the overall stability of the sample. This is conducive to improve the overall stability.

The results of the study will serve as a reference for the practical application of glass fiber in subgrade improvement.

## Figures and Tables

**Table 1:** Basic physical and mechanical parameters of aeolian sand.

Maximum Dry Density/(g·cm^−3^)	Relative Density	Liquid Limit/%	Plastic Limit/%	Plasticity Index	Cohesion/kPa	Internal Friction Angle/°
1.72	2.68	22.34	14.75	7.59	16.4	31.2

**Table 2:** Physical properties of glass fiber.

Fiber Type	$\mathrm{Density}/(g\cdot cm^{-3})$	Diameter/μm	Tensile Strength/MPa	Modulus of Elasticity/MPa	Fracture Strength/N·TEX-1
Glass fiber	2.5–2.7	9.3	280–310	3000–3600	0.65

**Table 3:** Orthogonal test factor levels.

Level	Factor		
	Fiber Content/‰	Fiber Length/mm	Cement Content/%
1	3	3	6
2	6	4.5	8
3	9	6	10

**Table 4:** Orthogonal test results.

Sample Number	Fiber Content‰	Fiber Length/mm	Cement Content/%	Splitting Strength/MPa
F1	3	3	6	0.15
F2	3	4.5	8	0.30
F3	3	6	10	0.51
F4	6	3	8	0.18
F5	6	4.5	10	0.29
F6	6	6	6	0.23
F7	9	3	10	0.21
F8	9	4.5	6	0.16
F9	9	6	8	0.19

**Table 5:** Range analysis outcomes.

Extreme Difference	Splitting Strength/MPa			
	Cement Content	Fiber Length	Fiber Content	Blank Column
K1	0.18	0.180	0.31	0.210
K2	0.22	0.250	0.23	0.24
K3	0.34	0.310	0.19	0.28
R	0.16	0.13	0.12	0.07

**Table 6:** Control variable test plan.

Sample Number	Cement Content/%	Fiber Content/‰	Fiber Length/mm
S1	10	2	3
S2	10	2	4.5
S3	10	2	6
S4	10	3	3
S5	10	3	4.5
S6	10	3	6
S7	10	4	3
S8	10	4	4.5
S9	10	4	6
S10	10	6	3
S11	10	6	4.5
S12	10	6	6
S13	10	0	0

**Table 7:** Reinforcement effect coefficient of splitting strength of specimens.

Fiber Length/mm	Fiber Content/‰			
	2	3	4	6
3	6.45	29.03	12.90	0
4.5	16.13	45.16	22.58	−9.68
6	48.39	61.29	45.16	−12.90

## References

[1] Fating Y. (2021). The theoretical implication of Xi Jinping’s ecological civilization thought. J. Grad. Sch. Chin. Acad. Soc. Sci..

[2] Gedela S.K., Subhani S.M., Bahurudeen A. (2021). Cleaner production of concrete by using industrial by-products as fine aggregate: A sustainable solution to excessive river sand mining. J. Build. Eng..

[3] Jianxun P. (2020). Study on Mechanical Properties of Fiber Reinforced Soil.

[4] Orakoglu M.E., Liu J. (2017). Effect of freeze–thaw cycles on triaxial strength properties of fiber-reinforced clayey soil. KSCE J. Civ. Eng..

[5] Patel S.K., Singh B. (2017). Strength and deformation behavior of fiber-reinforced cohesive soil under varying moisture and compaction states. Geotech. Geol. Eng..

[6] Roustaei M., Eslami A., Ghazavi M. (2015). Effects of freeze–thaw cycles on a fiber reinforced fine grained soil in relation to geotechnical parameters. Cold Reg. Sci. Technol..

[7] Xu J. (2021). Discrete Element Biaxial Test Simulation and Microscopic Characteristics of Glass Fiber Reinforced Soil.

[8] Ramkrishnan R., Sruthy M.R., Sharma A., Karthik V. (2018). Effect of random inclusion of sisal fibres on strength behavior and slope stability of fine grained soils. Mater. Today Proc..

[9] Ghorbani A., Salimzadehshooiili M., Medzvieckas J., Kliukas R. (2018). Strength characteristics of cement-rice husk ash stabilised sand-clay mixture reinforced with polypropylene fibers. Baltic J. Road Bridge Eng..

[10] Yang B.H., Weng X.Z., Liu J.Z. (2017). Strength characteristics of modified polypropylene fiber and cement-reinforced loess. J. Cent. South Univ..

[11] Dezheng R., Tang C.S., Hao C., Qing C., Haoda L., Bin S. (2021). Evaporation process and tensile strength characteristics of fiber reinforced adobe. J. Geotech. Eng..

[12] Yadav J.S., Tiwari S.K. (2016). Behaviour of cement stabilized treated coir fibre-reinforced clay-pond ash mixtures. J. Build. Eng..

[13] Tao P.H., Yongjing M., Tingwu L., Suping Y., Xinping Z. (2000). Effect of glass fiber on compressive and tensile strength of cement-soil. J. China Agric. Univ..

[14] Qun L., Shaolong G., Minmin W. (2016). Experimental study on mechanical properties of fiber cement soil. Geotech. Mech..

[15] Qian Y. (2019). Experimental Study on Tensile Properties of Fiber Reinforced Soil.

[16] Hengchao J., Qinglin L., Zhiyong Y. (2019). Experimental study on splitting tensile strength of glass fiber cement modified soil. Railway Sci. Eng. J..

[17] Hong S., Kaifeng J., Xueping W., Xiurun G. (2014). Glass fiber reinforced soil reinforcement technology for soft embankment. J. Undergr. Space Eng..

[18] Bo R., Chenxi R., Linfei D. (2021). Experimental study on tensile and compressive properties of polypropylene fiber reinforced cement mixed soil. Railway Sci. Eng. J..

[19] Bo R., Fen D., Wei D. (2021). Experimental study on triaxial compression of polypropylene fiber reinforced cement mortar soil. Railway Sci. Eng. J..

[20] Wei X., Junzhong L., Jun Z. (2021). Experimental study on durability of glass fiber reinforced cement soil. J. Railw. Sci. Eng..

[21] Chaosheng T., Bin S., Kai G. (2011). Microscopic study on the interaction between reinforcement and soil interface in fiber reinforced soil. Eng. Geol..

[22] Deyin W., Jian T.C.L., Baosheng L., Wei T., Kun Z. (2013). Shear strength characteristics of fiber reinforced unsaturated cohesive soil. Geotech. Eng. J..

[23] Lei G., Guohui H., Chen Y., Chao X., Junyi F., Nan X. (2016). Shear strength characteristics of basalt fiber reinforced clay. Chin. J. Geotech. Eng..

[24] Tang C.S., Li J., Wang D., Shi B. (2016). Investigation on the interfacial mechanical behavior of wave-shaped fiber reinforced soil by pullout test. Geotext. Geomembr..

[25] Tang C.S., Wang D., Cui Y.-J., Shi B., Li J. (2016). Tensile strength of fiber-reinforced soil. J. Mater. Civ. Eng..

[26] Shizong Z. (2017). Experimental Study on Strength Characteristics of Polypropylene Fiber Reinforced Cement Soil.

[27] Jinquan X. (2006). Interfacial Mechanics.

[28] Xiangdong Z., Jun L., Qi S., Fu Y., Jiashun L., Zhi Q. (2018). Study on negative temperature dynamic performance and rheological characteristics of cement-improved aeolian sand. Geotech. Mech..

[29] Zhilun X. (2016). Elastic Mechanics.

[30] Fatahi B., Khabbaz H., Fatahi B. (2012). Mechanical characteristics of soft clay treated with fibre and cement. Geosynth. Int..

